# Analgesic effects of a highly selective mPGES-1 inhibitor

**DOI:** 10.1038/s41598-023-30164-3

**Published:** 2023-02-27

**Authors:** Madeline J. Stewart, Lauren M. Weaver, Kai Ding, Annet Kyomuhangi, Charles D. Loftin, Fang Zheng, Chang-Guo Zhan

**Affiliations:** 1 Molecular Modeling and Biopharmaceutical Center, College of Pharmacy, University of Kentucky, 789 South Limestone Street, Lexington, KY 40536 USA; 2 Department of Pharmaceutical Sciences, College of Pharmacy, University of Kentucky, 789 South Limestone Street, Lexington, KY 40536 USA

**Keywords:** Chemical biology, Drug discovery

## Abstract

The growing opioid use and overdose crisis in the US is closely related to the abuse of pain medications. Particularly for postoperative pain (POP), ~ 310 million major surgeries are performed globally per year. Most patients undergoing surgical procedures experience acute POP, and ~ 75% of those with POP report the severity as moderate, severe, or extreme. Opioid analgesics are the mainstay for POP management. It is highly desirable to develop a truly effective and safe non-opioid analgesic to treat POP and other forms of pain. Notably, microsomal prostaglandin E2 (PGE_2_) synthase-1 (mPGES-1) was once proposed as a potentially promising target for a next generation of anti-inflammatory drugs based on studies in mPGES-1 knockouts. However, to the best of our knowledge, no studies have ever been reported to explore whether mPGES-1 is also a potential target for POP treatment. In this study, we demonstrate for the first time that a highly selective mPGES-1 inhibitor can effectively relieve POP as well as other forms of pain through blocking the PGE_2_ overproduction. All the data have consistently demonstrated that mPGES-1 is a truly promising target for treatment of POP as well as other forms of pain.

## Introduction

The US is suffering from a growing opioid use and overdose crisis, which is closely related to the abuse of pain medications. Particularly for postoperative pain (POP), ~ 51.4 million inpatient surgeries^1^ and ~ 129 million outpatient surgeries^2^ are performed in the US per year. Globally, ~ 310 million major surgeries are performed per year^3^. Most patients undergoing surgical procedures experience acute POP, and ~ 75% of those with POP report the severity as moderate, severe, or extreme^4^. Opioid analgesics are commonly used as the mainstay for POP management. POP is not adequately managed in > 80% of patients in the US, and poorly controlled acute POP is associated with increased morbidity, functional and quality-of-life impairment, delayed recovery time, prolonged duration of opioid use, and higher health-care costs^5^. It is exceedingly desirable to develop a truly effective and safe non-opioid analgesic to treat POP and other forms of pain. Here we show that a highly selective microsomal prostaglandin E2 synthase-1 (mPGES-1) inhibitor can effectively relieve multiple forms of pain, including POP. This is the first demonstration that an mPGES-1 inhibitor can effectively relieve POP. All the data have consistently validated mPGES-1 as a truly promising target for treatment of POP as well as other forms of pain. The potential of a highly selective mPGES-1 inhibitor as a substitute for currently used opioids is particularly significant for future postoperative pain management, as opioids are still the mainstay of postoperative analgesia because a truly effective and safe non-opioid analgesic is not available.

**Figure 1:**
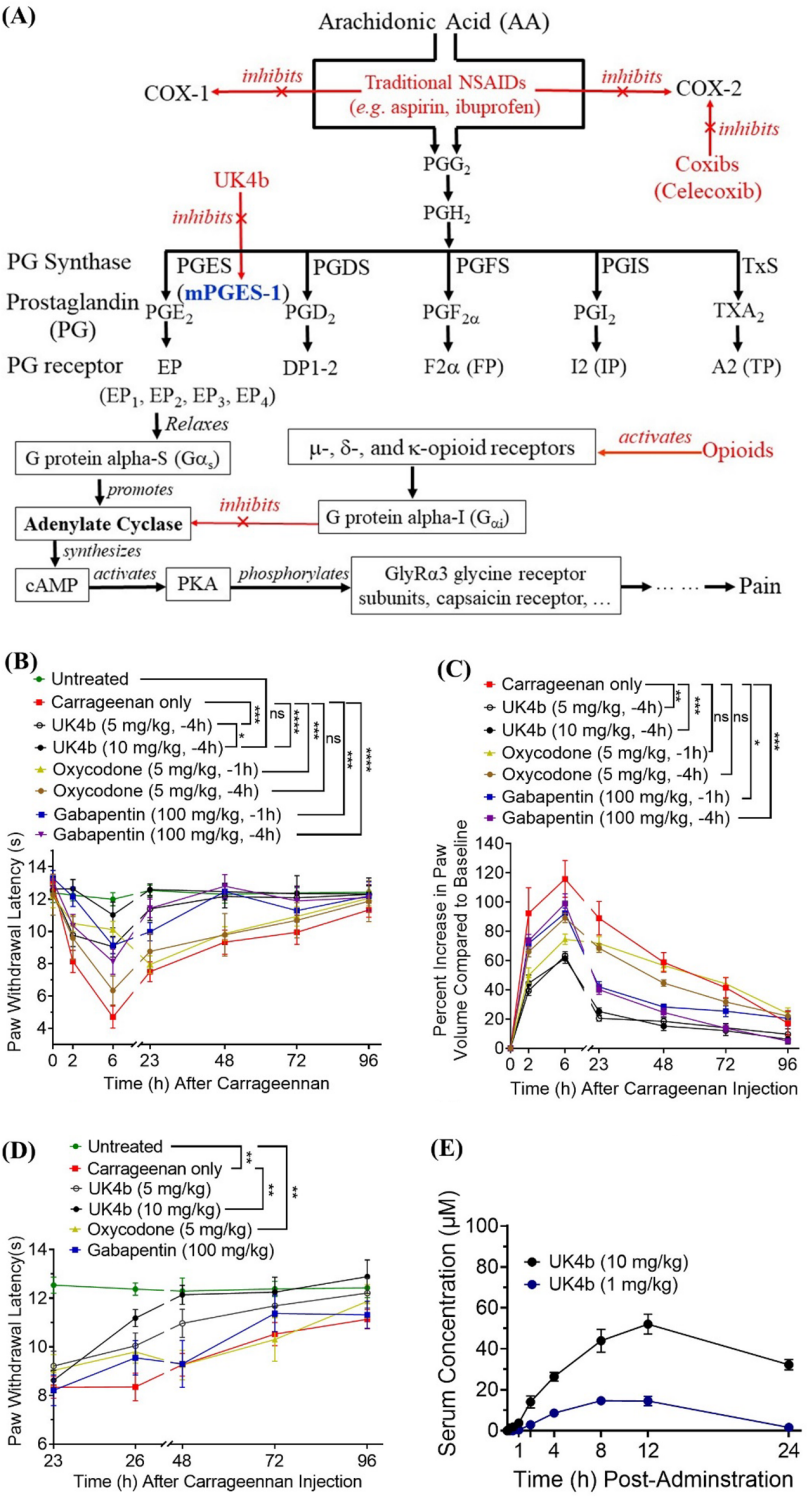
A schematic of the up and downstream processes of pain involving PGE_2_ (**A**), the anti-inflammatory and analgesic effects of UK4b on carrageenan-induced hyperalgesia (represented by PWL) and paw edema (represented by the percent increase in the paw volume), and the pharmacokinetics (PK) of UK4b in rats. Treatment (UK4b or oxycodone or gabapentin or vehicle, IP) was injected 4 h (or 1 h, for oxycodone/gabapentin only) before carrageenan injection. Hyperalgesia (**B**) and paw edema (**C**) were assessed with a pre-treatment model (n = 10 for each group). Hyperalgesia was also assessed in a post-treatment model (**D**). Carrageenan was administered to all groups. After the 23-h measurement, vehicle, UK4b (5 or 10 mg/kg), oxycodone (5 mg/kg), or gabapentin (100 mg/kg) were administered (IP) and PWL was assessed at 26, 48, 72, and 96 h relative to carrageenan administration (n = 10 for each group). Statistical significance (two-way ANOVA): *p < 0.05; **p < 0.01; ***p < 0.001; ****p < 0.0001. (**E**) Time-dependent concentrations of UK4b after IP injection of 1 mg/kg UK4b (n = 7) or 10 mg/kg UK4b (n = 4).

As is well known, opioids are a class of analgesics with the capacity to deliver pain relief by activating opioid receptors, particularly µ-opioid receptors. While effective as analgesics, opioids are also associated with abuse and physical dependence potential. Currently available opioids also have other side effects including constipation, sexual dysfunction, and depression^6^. The other major class of analgesics in clinical use are nonsteroidal anti-inflammatory drugs (NSAIDs) that inhibit cyclooxygenase (COX) enzymes, including both COX-1 and COX-2. COX enzymes are necessary for biosynthesis of prostaglandin E2 (PGE_2_, which is a prostanoid serving as the principal pro-inflammatory mediator) as shown in Fig. 1A, and these drugs are not associated with abuse potential. However, traditional NSAIDs (COX-1/2 inhibitors) have significant cardiovascular, cerebrovascular, and gastrointestinal risks that have limited their utilization^7,8^. The remaining classes of medications used for pain management, including antidepressants and anticonvulsants, provide limited pain relief and/or various side effects as well^9^. So, a truly safe, effective and non-addictive analgesic is an unmet medical need.

Particularly for POP, opioids are recognized as having unrivaled analgesic efficacy^10^. Their adverse effects including addiction led to the search for better options. Many potential targets have been explored^10^, but mPGES-1 is not one of them. In this study, we hypothesize that mPGES-1 is a more promising target for treatment of POP in terms of both efficacy and safety due to the following mechanistic background.

Generally speaking, pain can start from tissue damage/injury and the corresponding inflammatory response, and the two major classes of pain medications are closely related in the primary mechanism of pain. Specifically, tissue damage induces release of prostaglandin E2 (PGE_2_)—the principal pro-inflammatory mediator^11–14^. The PGE_2_ biosynthesis^15^ starts from arachidonic acid (AA). COX-1 and 2 convert AA to prostaglandin H2 (PGH_2_)^15^, and prostaglandin E synthase (PGES) transforms PGH_2_ to PGE_2_ (see Fig. 1A) ^16^. Notably, PGH_2_ is a common substrate for multiple synthases responsible for production of five prostanoids including prostacyclin (PGI_2_), prostaglandin D_2_ (PGD_2_), prostaglandin F_2α_ (PGF_2α_), and thromboxane A_2_ (TXA_2_), in addition to PGE_2_^17^. It is known that the PGES enzymes responsible for the biosynthesis of PGE_2_ include microsomal PGES-1 (mPGES-1), mPGES-2, and cytosolic PGES (cPGES, mainly responsible for producing basal level of PGE_2_ for physiological homeostasis^18,19^). Within the three enzymes, only mPGES-1 is highly inducible; it is only weakly expressed under the normal physiological conditions, and upregulated strongly in the inflammatory state. Enzyme cPGES and mPGES-2 are constitutively expressed^20^, and are only responsible for the production of basal level of PGE_2_. So, selective inhibition of mPGES-1 will not shut down the production of basal level of PGE_2_ required for physiological homeostasis.

As indicated in Fig. 1A, PGE_2_ acts on EP receptors (particularly EP_2_) expressed by excitatory interneurons and projection neurons in the superficial dorsal horn and stimulates adenylate cyclase—an enzyme producing the intracellular second messenger, 3′-5′-cyclic adenosine monophosphate (cAMP)^21^. Resultant stimulation of the cAMP-protein kinase A (PKA) pathway phosphorylates GlyRα3 glycine receptor subunits etc., rendering the neurons unresponsive to the inhibitory effects of glycine^21,22^. As indicated in Fig. 1A, an opioid activates opioid receptors that eventually inhibit adenylate cyclase and, thus, decreases the cAMP signaling^23^. So, the two classes of pain medications (NSAIDs and opioids) target different stages of the primary physiological process of pain. NSAIDs target the upstream process of pain (PGE_2_ production), whereas opioids target the downstream process of pain (one of the consequences of the promoted production of PGE_2_). The upstream and downstream processes cross at the adenylate cyclase activity (see Fig. 1A): whereas NSAIDs may lead to effective prevention of the adenylate cyclase activity promotion, opioids may lead to effective inhibition of the adenylate cyclase activity. Over inhibition of the adenylate cyclase activity may be associated with serious adverse effects. So, in principle, both NSAIDs and opioids may eventually and effectively attenuate the adenylate cyclase activity and, thus, relieve pain through decreasing the adenylate cyclase activity. But they are expected to have different types of adverse effects.

The first generation of NSAIDs, such as ibuprofen (IC_50_ = 13 μM against COX-1 and IC_50_ = 370 μM against COX-2)^24^, are weak inhibitors of both COX-1 and 2. The second generation of NSAIDs, known as Coxibs, including celecoxib, rofecoxib, and valdecoxib, selectively inhibit COX-2 with a low-nanomolar IC_50_. However, these COX-2 specific inhibitors have various serious side effects, such as increasing the risk of fatal heart attack or stroke and causing stomach or intestinal bleeding. The serious side effects, which led to withdrawal of rofecoxib and valdecoxib (although celecoxib remains in clinical use), are due to the fact that the synthesis of all other physiologically required prostanoids (PGD_2_, PGF_2α_, PGI_2_, and TXA_2_, as shown in Fig. 1A) downstream of PGH_2_ are blocked by COX-1/2 inhibition. For example, blocking the production of PGI_2_ will cause significant cardiovascular problems^25^. So, clinical use of a truly potent COX-2 inhibitor such as celecoxib (IC_50_ = 40 nM against COX-2) at a sufficiently effective dose must be associated with serious side effects. As an inducible enzyme, mPGES-1 should be a more promising, potentially ideal target for anti-inflammatory pain relief, because the mPGES-1 inhibition will only block the mPGES-1-catalyzed PGE_2_ over-production without blocking the normal production of PGE_2_ and other prostanoids (Fig. 1A). Hence, it is reasonable to hypothesize that an mPGES-1 inhibitor (which directly inhibits the over-production of PGE_2_) can be expected to retain the anti-inflammatory and analgesic effects of COX-1/2 inhibitors, but with less side effects caused by the COX-1/2 inhibition.

In fact, numerous inhibitors of human mPGES-1 have been reported in literature. However, most of the discovered human mPGES-1 inhibitors are inactive against mouse or rat mPGES-1^26^, because most of the reported mPGES-1 inhibitors bind in a non-conserved region of the active site cavity with huge differences between human and mouse/rat^27^. Only modest anti-inflammatory effects of mPGES-1 inhibitors have been demonstrated in literature so far. As a result, mPGES-1 as a potentially promising and practically feasible target for effective pain relief still remains to be validated. Nevertheless, in a recently reported effort targeting a conserved region of the mPGES-1 active site^27^, we were able to design and discover a novel type of inhibitor, compound 4b (denoted as UK4b for convenience) potent against both human and mouse mPGES-1 enzymes as a chemical probe (IC_50_ = 33 nM against human mPGES-1 and IC_50_ = 157 nM against mouse mPGES-1)^27^. It was also demonstrated that UK4b was highly selective for mPGES-1 over COX-1/2 and that UK4b effectively decreased carrageenan-stimulated PGE_2_ over-production^27^. However, it is unknown whether UK4b has the desirable anti-inflammatory and analgesic effects. In this report, we demonstrate the analgesic effects of UK4b for the first time.

## Results

### Anti-inflammatory and analgesic effects of UK4b on carrageenan-induced paw edema and hyperalgesia

In the present study, we first tested UK4b in the wild-type rat model of carrageenan-induced paw edema and hyperalgesia (Fig. 1B,C) in comparison with oxycodone (a representative strong opioid for pain relief) and gabapentin (the most representative anticonvulsant for pain relief). For oxycodone tablets, the FDA-approved maximum dose for first-time users is 40 mg; single doses higher than 40 mg are only for use in opioid-tolerant patients^28^. The rat dose corresponding to an human equivalent dose (HED) of 40 mg (for an average human body weight of 60 kg) is (40/60) × 6.3 = 4.2 mg/kg, according to the generally accepted animal–human dose conversion guide^29^. For gabapentin tablets, the FDA-approved maximal dose for a new patient is 300 mg up to three times per day, or 900 mg/day^30^. The rat dose that corresponds to the HED of 900 mg is (900/60) × 6.3 = 94.5 mg/kg. So, we elected to use 5 mg/kg oxycodone and 100 mg/kg gabapentin in our tests to compare with 5 and 10 mg/kg UK4b. In this animal model, the hyperalgesia/pain is reflected by the paw withdrawal latency (PWL). The shorter the PWL time, the more severe the pain. As shown in Fig. 1, carrageenan induced hyperalgesia (Fig. 1B) and edema (which is reflected by the percent increase in the paw volume) (Fig. 1C) in rats for about four days, in the carrageenan control group (treated with vehicle). Pretreatment with UK4b effectively and dose-dependently reduced the hyperalgesia (Fig. 1B) and decreased the edema (Fig. 1C). Particularly for hyperalgesia, pretreatment with 10 mg/kg UK4b completely suppressed carrageenan-induced hyperalgesia. There was no significant difference in PWL between the 10 mg/kg UK4b treatment group and the control group (untreated rats without pain at all). In comparison, gabapentin similarly relieved the hyperalgesia, but 100 mg/kg gabapentin was not as effective as 10 mg/kg UK4b. Oxycodone also effectively relieved the pain within six hours after carrageenan injection, but there was still significant pain at and after 23 h. So, 10 mg/kg UK4b was more effective than 5 mg/kg oxycodone and 100 mg/kg gabapentin in terms of the anti-inflammatory and analgesic effects.

To better model the clinical treatment of pain, we also tested UK4b in a posttreatment model of carrageenan-induced hyperalgesia. Rats were first injected with carrageenan to induce the hyperalgesia/pain. On the next day (23 h later), there was still persistent pain (see Fig. 1D for vehicle control group—red line), allowing us to test the posttreatment effects of UK4b in comparison with oxycodone and gabapentin. So, at 23 h after the carrageenan injection, the rats were treated with UK4b, oxycodone, gabapentin, or vehicle (control), after the 23-h PWL measurement occurred. As shown in Fig. 1D, administration of 5 mg/kg oxycodone or 100 mg/kg gabapentin significantly relieved the pain, as expected. UK4b also significantly, and dose-dependently, relieved the pain. However, unlike with oxycodone and gabapentin, UK4b persistently resulted in higher PWL. Overall, in both the pre- and post-treatment models (Fig. 1B–D), the anti-inflammatory and analgesic effects of UK4b are superior compared to both oxycodone and gabapentin.

### Anti-inflammatory and analgesic effects of UK4b on adjuvant-induced knee joint arthritis

**Figure 2:**
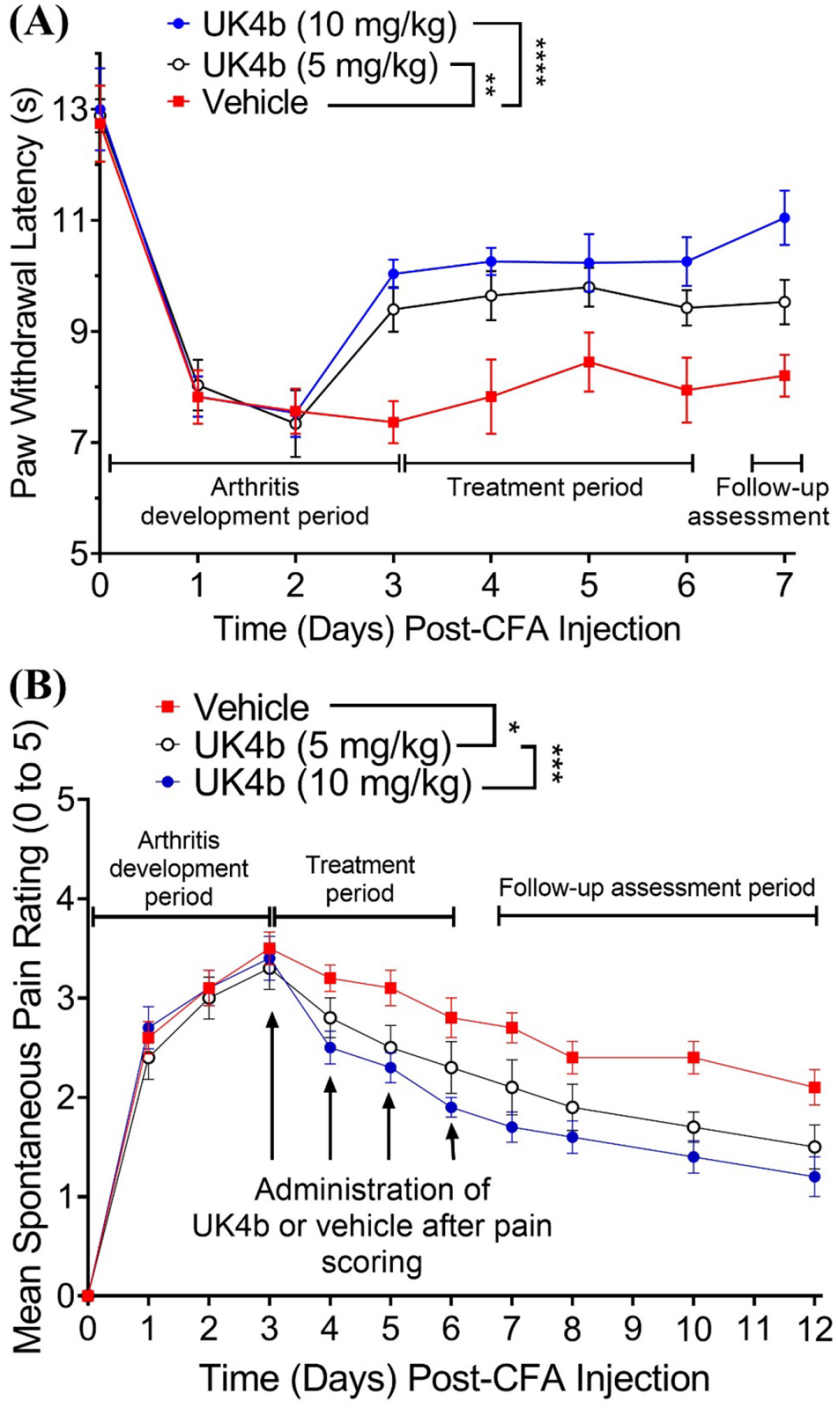
Anti-inflammatory and analgesic effects of UK4b on CFA-induced knee joint arthritis in rats (n = 10 for each group). CFA was injected to the knee joint on Day 0. UK4b (5 or 10 mg/kg) or vehicle (control) was injected (IP) daily on Days 3 to 6. (**A**) PWL data measured on Days 0 to 7. (**B**) Arthritis score (mean spontaneous pain rating) measured on Days 0 to 8, 10, and 12. Statistical significance (two-way ANOVA): *p < 0.05; **p < 0.01; ***p < 0.001; ****p < 0.0001.

UK4b was further tested using the Complete Freund’s Adjuvant (CFA)-induced knee joint arthritis model described by Hammell et al.^31^ Using this one-week knee joint arthritis model, CFA was injected to the knee joint on Day 0 to induce chronic arthritis and associated pain, followed by daily PWL and arthritis score (mean spontaneous pain rating) assessments from Day 0 to Day 7. Daily treatment with UK4b (5 or 10 mg/kg) or vehicle (control group) started on Day 3 (after the arthritis score assessment but before the PWL assessment on that day) and continued until Day 6. So, there were a total of four doses of the respective treatment from Day 3 to Day 6. After the CFA injection on Day 0, the PWL rapidly decreased (Fig. 2A) while the arthritis score rapidly increased (Fig. 2B) beginning Day 1. On Day 3, the arthritis score reached the peak (reflecting the most severe arthritis), while PWL reached the minimum (reflecting the most severe pain) for rats in the vehicle control group, which is consistent with previous observations reported by Hammell et al. for the control group^31^.

As seen in Fig. 2A, compared to the vehicle control group, the UK4b treatment dose-dependently relieved the pain during the treatment days (Days 3 to 6), as expected. Interestingly, rats in the UK4b groups continued to have significantly less pain on Day 7 even without further treatment with UK4b on the day. So, the daily treatment with UK4b during Days 3 to 6 continued to relieve the pain beyond the treatment period. In addition, compared to the vehicle control group, the UK4b treatment also significantly and dose-dependently accelerated the decrease of the arthritis score, as shown in Fig. 2B. We also assessed the arthritis score at three more time points (Days 8, 10, and 12) as further follow-up assessment beyond the one-week period of the standard adjuvant-induced knee joint arthritis model described by Hammell et al.^31^ On Day 12 (the last day of the follow-up assessment period), the average arthritis score for the vehicle control group was 2.1 for the vehicle control group, 1.5 for the 5 mg/kg UK4b treatment group, and 1.2 for the 10 mg/kg UK4b treatment group, demonstrating the dose-dependent anti-inflammatory effect of UK4b.

### Analgesic effects of UK4b on postoperative pain

**Figure 3:**
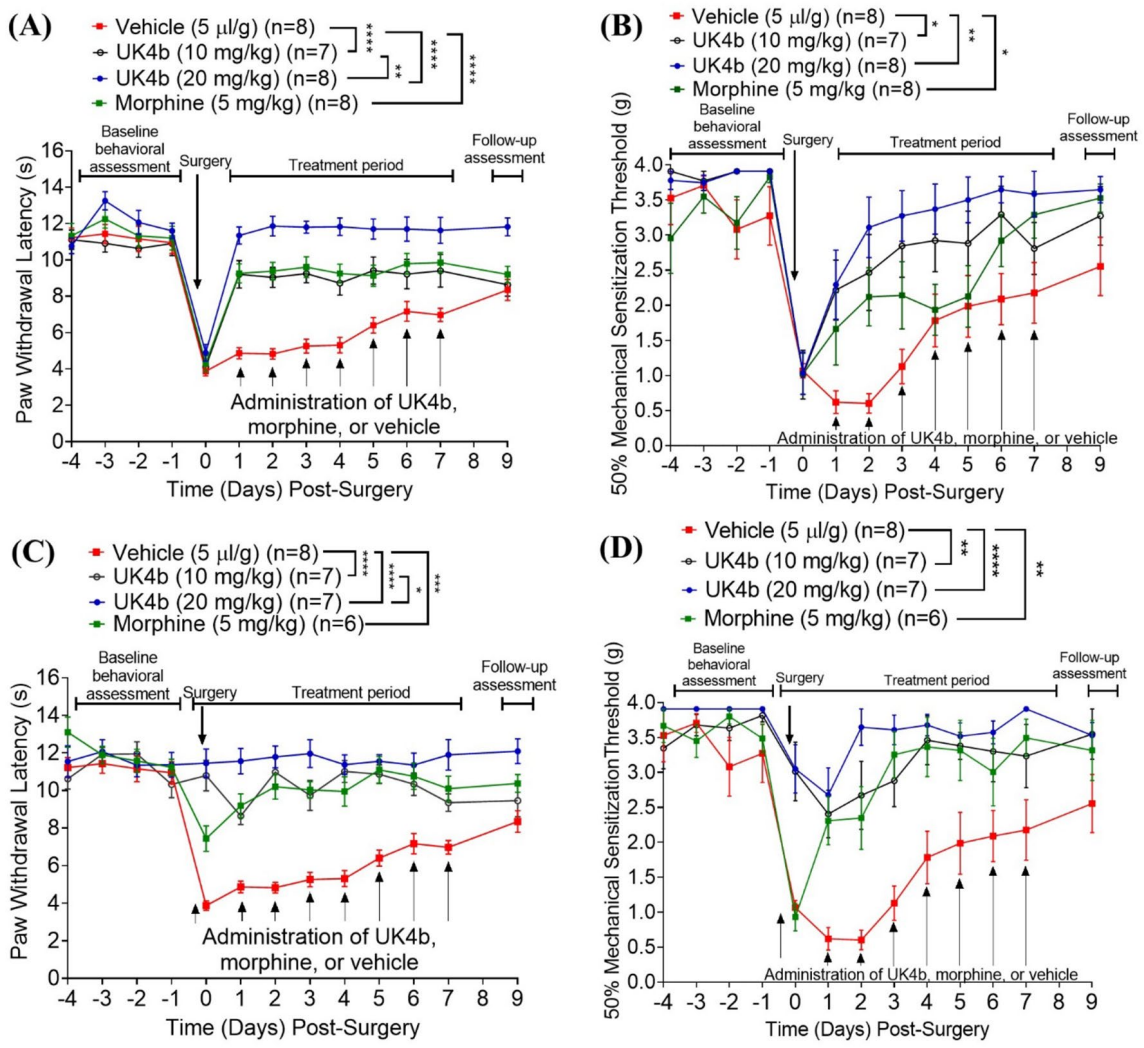
The analgesic effects of UK4b (10 or 20 mg/kg) compared to vehicle control and morphine (5 mg/kg) in the mouse model of postoperative pain (n ≥ 6 for each group). All treatments were administered subcutaneously (SC). (**A**) Paw withdrawal latency (PWL) and (**B**) 50% mechanical sensitization threshold (grams) in the post-treatment model, with surgery occurring on Day 0 and daily treatment from Days 1 to 7. (**C**) Paw withdrawal latency (PWL) and (**C**) 50% mechanical sensitization threshold (grams) in the pre- and post-treatment model, with surgery occurring on Day 0 and daily treatment from Days 0 to 7. PWL and mechanical sensitization were measured at 2 h after surgery (Day 0) or 2 h after each daily treatment with UK4b or morphine or vehicle (Days 1 to 7). Statistical significance (two-way ANOVA): *p < 0.05; **p < 0.01; ***p < 0.001; ****p < 0.0001.

With the encouraging analgesic effects of UK4b in the aforementioned animal models, we also examined UK4b for its analgesic effects on postoperative pain in comparison with morphine (one of the popularly used strong opioids for postoperative pain relief in clinical practice) using an incisional model of postoperative pain in mice^32^. The incisional surgery on Day 0 was followed by daily pain treatment for seven days (Days 1 to 7) after the surgery, with an additional follow-up assessment on Day 9 (Fig. 3A,B). Pain was analyzed daily by measuring the PWL (hyperalgesia) and 50% mechanical sensitization threshold (allodynia) for (see Methods section, SI for the details).

As an injectable for postoperative analgesia, morphine can be administered intramuscularly (IM), intravenously (IV), subcutaneously (SC)^33^ or intraperitoneally (IP)^34^. The clinically used range of morphine doses as an injectable for postoperative analgesia is 0.03–0.2 mg/kg^33^. Dose of 0.2 mg/kg for humans is associated with a mouse dose of 0.2 × 12.3 = 2.5 mg/kg according to the generally accepted animal–human dose conversion guide^29^. Notably, a range of morphine dose levels were used by Curtin et al. in studying postoperative pain relief in rodents, with the highest dose of morphine being 5 mg/kg^35^. Hence, we used 5 mg/kg morphine/day as a control in our tests.

As seen in Fig. 3A,B (also Fig. S1 in Supplementary Information), compared to the baseline PWL and 50% mechanical threshold (as the average during Day -4 to -1 before the surgery), mice in all groups had similar hyperalgesia (reflected by the lower PWL) and allodynia (reflected by the lower 50% mechanical threshold). Daily treatment with 5 mg/kg morphine beginning Day 1 significantly attenuated both the postoperative hyperalgesia and allodynia, as expected. Interestingly, UK4b also significantly (and dose-dependently) attenuated both the postoperative hyperalgesia and allodynia. Remarkably, daily treatment with 20 mg/kg UK4b completely blocked the postoperative hyperalgesia (Fig. 3A) and was much more effective in attenuating the postoperative allodynia (Fig. 3B) compared to the daily treatment with 5 mg/kg morphine.

Further, we also repeated the tests with the incisional surgery, but with an additional dose of UK4b, morphine, or vehicle on Day 0 at -2 h (two hours before the surgery). As seen in Fig. 3C,D, the pretreatment with UK4b (10 or 20 mg/kg UK4b) also completely blocked the postoperative hyperalgesia (Fig. 3C) while effectively attenuating the postoperative allodynia (Fig. 3D) on Day 0. In comparison, the pretreatment with morphine did not significantly attenuate the postoperative allodynia on Day 0 (Fig. 3D), while significantly attenuating the postoperative hyperalgesia on Day 0 (Fig. 3C).

### Pharmacokinetics and pharmacodynamics of UK4b

It is important to know whether the observed analgesic effects of UK4b are consistent with its pharmacokinetics (PK) and pharmacodynamics (PD). As is well-known, the PK profile of a compound is dependent on its fundamental absorption, distribution, metabolism, and excretion (ADME) properties. Particularly, the biological half-life of a compound is closely related to its liver microsomal stability (which is affected by all metabolic enzymes in the liver microsomes). Hence, the intrinsic clearance of UK4b in liver microsomes (human) was determined by Eurofins service, demonstrating satisfactory stability in human liver microsomes (*t*_1/2_ > 72 min and CL_int_ < 0.00963 μL/min/mg). In addition, through further service by Eurofins, we also examined the potential inhibitory activity of UK4b at 10 μM against potassium channel hERG, showing insignificant (< 5%) inhibition at 10 μM.

According to the determined time-dependent serum concentrations of UK4b shown in Fig. 1E, after administration of 10 mg/kg UK4b, the serum UK4b concentration was still significant at 24 h, which is consistent with its microsomal stability and long-lasting analgesic effects.

It is also interesting to know whether the analgesic effects of UK4b are associated with its effects on the PGE_2_ concentrations. In theory, it would be most convenient to measure the PGE_2_ concentrations in plasma samples. However, PGE_2_ has a very short plasma half-life (2.5–5 min)^36^ such that it would be challenging to accurately measure the plasma PGE_2_ concentrations. Nevertheless, we were able to collect relevant paw tissue from carrageenan-induced edema and hyperalgesia in rats (at 26 h after the carrageenan injection) and the paw tissue from the mice with the incisional surgery (at 48 h after the surgery) and determine their PGE_2_ concentrations by using an ELISA assay, although we were not able to collect relevant knee joint tissue from CFA-injected rats.

**Figure 4:**
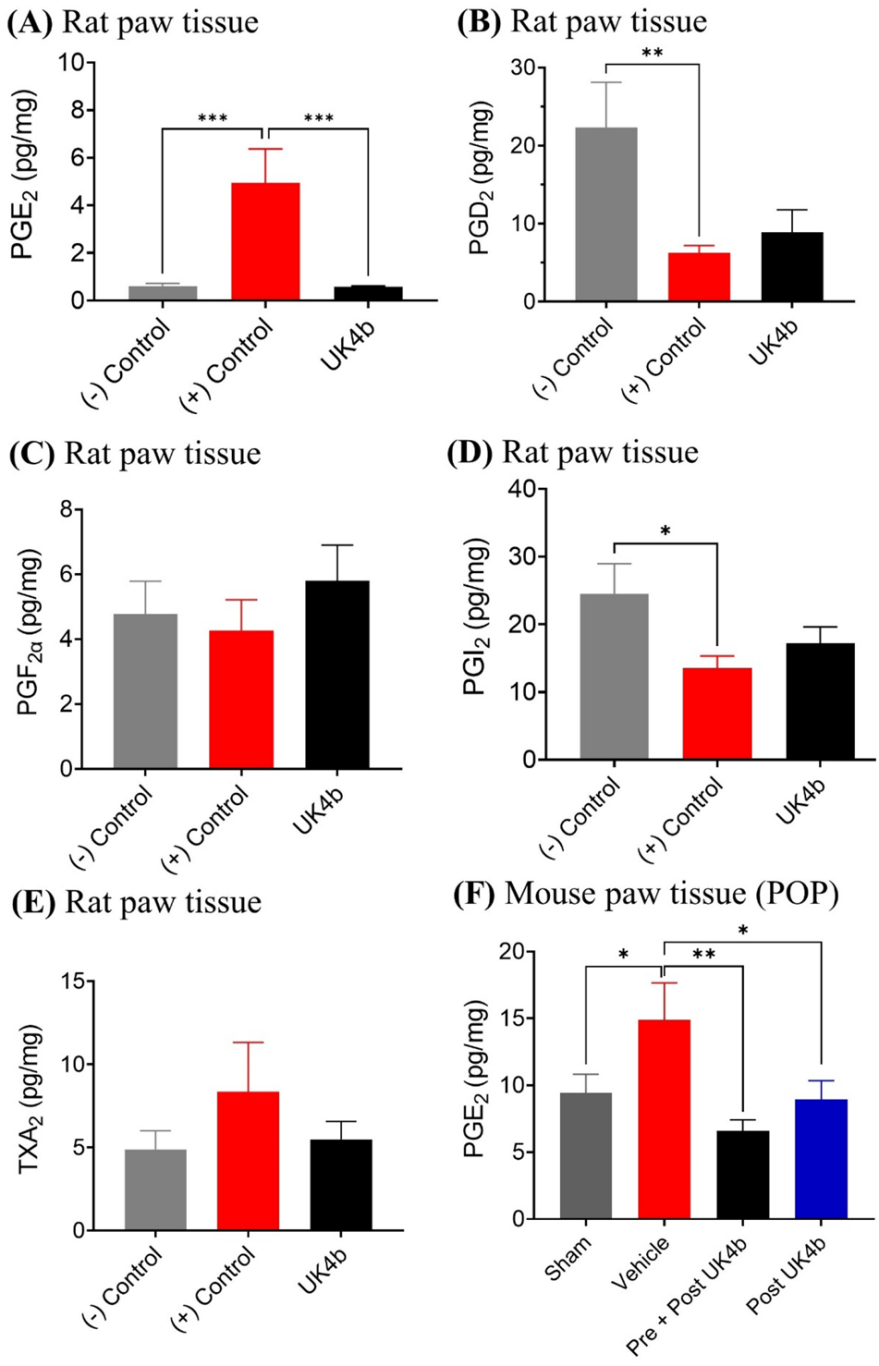
The effects of UK4b on the concentrations (pg/mg) of prostanoids (PGE_2_, PGD_2_, PGF_2α_, PGI_2_, TXA_2_) in tissues. (**A**) PGE_2_, (**B**) PGD_2_, (**C**) PGF_2α_, (**D**) PGI_2_, (**E**) TXA_2_ concentrations in paw tissue collected from carrageenan-injected rats at 26 h after the carrageenan injection: the (−) control group (without carrageenan injection) provides the baseline concentrations, the (+) control group received carrageenan injection and was treated with vehicle, and the UK4b group was treated with 10 mg/kg UK4b (IP) 4 h before carrageenan injection (n ≥ 4 for each group). (**F**) PGE_2_ concentrations in the paw tissue collected at 48 h (Day 2) from four groups of mice after the surgery in the model of postoperative pain: Sham–sham surgery group; vehicle—surgery group treated with vehicle; Pre + post UK4b—pre- and post-treatment group received daily treatment with 20 mg/kg UK4b from Days 0 (2 h before surgery) to 2; and Post UK4b—post-treatment group treated with 20 mg/kg UK4b on Days 1 and 2 (n ≥ 6 for each group). Statistical significance (one-way ANOVA): *p < 0.05; **p < 0.01; ***p < 0.001; ****p < 0.0001.

As shown in Fig. 4A, without carrageenan injection, the baseline PGE_2_ concentration in the paw tissue was 0.61 pg/mg in average. The average PGE_2_ concentration increased to 4.9 pg/mg in the carrageenan control group—an eightfold increase. Treatment with 10 mg/kg UK4b effectively blocked the PGE_2_ overproduction. As a result, there was no difference in the average PGE_2_ concentration between the vehicle control rats (no carrageenan injection, 0.61 pg/mg) and 10 mg/kg UK4b-treated rats (0.58 pg/mg) after carrageenan injection. The relative magnitudes of the measured PGE_2_ concentrations in different groups are consistent with the PWL data shown in Fig. 1B. Additionally, we also determined the concentrations of all other prostanoids (PGD_2_, PGF_2α_, PGI_2_, and TXA_2_) by using ELISA assays with the corresponding antibodies. As shown in Fig. 4 (panels B to E), the treatment with 10 mg/kg UK4b did not significantly change any of the PGD_2_, PGF_2α_, PGI_2_, and TXA_2_ concentrations, while significantly blocking the PGE_2_ overproduction (Fig. 4A). For this reason, we only determined the PGE_2_ concentrations in the mouse model of postoperative pain.

According to the PGE_2_ data shown in Fig. 4F, mice in the sham surgery group have an average PGE_2_ concentration of 9.4 pg/mg. The average PGE_2_ concentration increased to 15 pg/mg in the surgery group treated with vehicle—an ~ twofold increase at 48 h after the surgery. The pre- and/or post-treatment with UK4b all significantly lowered the PGE_2_ concentration compared to the surgery group treated with vehicle. There was no significant difference in the average PGE_2_ concentration between the sham surgery group and the UK4b-treated groups after the surgery. So, the relative magnitudes of the measured PGE_2_ concentrations in different groups are consistent with the PWL and 50% mechanical threshold data shown in Fig. 3.

## Discussion

The contribution of mPGES-1 in numerous pathological conditions has been studied using mPGES-1 knockouts in a variety of mouse models of inflammation-related diseases^25,37–44^. Particularly, the first study on pathogenesis of collagen-induced arthritis^38^ demonstrated that, in contrast to COX-2 knockouts, mPGES-1-deficient mice are viable, fertile and have no abnormal physiological phenotypes, but have markedly reduced inflammatory responses and hyperalgesia. In further reports, mPGES-1 was proposed as a potentially promising target for the treatment of many other inflammation-related diseases such as osteoarthritis, rheumatoid arthritis and associated pain, cardiovascular diseases, and cancer^25,37–44^. However, to the best of our knowledge, no study has ever been reported to explore whether mPGES-1 is also a potentially promising target for treatment of postoperative pain or not. Further, even for pain conditions other than postoperative pain or other inflammation-related diseases, the actual value of mPGES-1 as a drug target is still vague, because there has been no report demonstrating that administration of a selective mPGES-1 inhibitor has comparable effects to the mPGES-1 gene deletion in wild-type rodents. There are at least two possibilities why the encouraging effects of the mPGES-1 gene deletion for inflammation and associated pain in mice have not been demonstrated by administration of a selective mPGES-1 inhibitor to wild-type mice in any pain model. First, it could be due to the inherent difference between the mPGES-1 gene deletion (which is equivalent to the long-lasting 100% inhibition of mPGES-1) and the practical mPGES-1 inhibition (partial and transient mPGES-1 inhibition). Is it necessary to 100% inhibit mPGES-1 in order for a selective mPGES-1 inhibitor to be similarly effective? Another possible reason is the mPGES-1 inhibitors tested so far might not be sufficiently potent or may have other possible problems such that none of these mPGES-1 inhibitors can serve as a good chemical probe to validate mPGES-1 as a truly promising target for treatment of inflammation and related diseases.

In this study, UK4b, a highly selective mPGES-1 inhibitor serving as a novel chemical probe, has been tested in rodent models of carrageenan-induced edema and hyperalgesia, CFA-induced knee joint arthritis, and postoperative pain. In the rat models of carrageenan-induced edema and hyperalgesia, our data revealed that UK4b more effectively attenuated the pain than both oxycodone and gabapentin in a dose-dependent manner through selectively blocking the carrageenan-induced PGE_2_ overproduction without significantly changing the concentrations of all other prostanoids (PGD_2_, PGF_2α_, PGI_2_, and TXA_2_) in the tissue. UK4b was also able to significantly and dose-dependently attenuate CFA-induced knee joint arthritis and associated pain. Additionally, in the mouse model of postoperative pain, UK4b also effectively attenuated postoperative pain, including both the hyperalgesia and allodynia, in a dose-dependent manner through effectively blocking the surgery-induced PGE_2_ overproduction, and the data suggests that UK4b might be more effective than morphine in the postoperative pain relief. Further, it should be noted that UK4b is ~ fivefold more potent (IC_50_ = 33 nM) against human mPGES-1 than its inhibitory activity against mouse mPGES-1 (IC_50_ = 157 nM)^27^. Hence, it is reasonable to assume that UK4b could be even more effective for pain relief in humans. Our findings in this study have favorably validated mPGES-1 as a truly promising target for effective treatment of various forms of pain, including postoperative pain.

There has been an open question^45,46^ concerning whether the mPGES-1 inhibition will indirectly increase the production of other prostanoids while decreasing the PGE_2_ production and whether the mPGES-1 inhibition will significantly decrease the basal level of PGE_2_ production. The tissue prostanoid data obtained in this study suggest that mPGES-1 inhibition will only significantly block the PGE_2_ overproduction without significantly increasing the production of other prostanoids. The PGE_2_ concentrations in the UK4b treatment groups are all comparable to the corresponding PGE_2_ concentration baselines, implying that the mPGES-1 inhibitor only inhibits the PGE_2_ overproduction without blocking the normal production of the basal level of PGE_2_ and, hence, the mPGES-1 inhibition should be safe.

Further, based on the comparison of UK4b with opioids in carrageenan-induced hyperalgesia and postoperative pain, it is reasonable to conclude that a highly selective mPGES-1 inhibitor could be as effective as, or even more effective than, a strong opioid in the treatment of pain including postoperative pain. Hence, a highly selective mPGES-1 inhibitor may serve as a substitute for currently used opioids in the treatment of postoperative pain and other forms of pain. Such a substitute is expected to be safer and non-addictive, without the serious adverse effects of opioids or traditional NSAIDs associated with the inhibition of COX-1/2. In fact, our previous study^27^ demonstrated that UK4b is much safer than celecoxib for use in mice. Administration of UK4b at extremely high doses (1 or 5 g/kg) in mice did not cause any toxicity signs in animal behavior and in microscopic histological evaluation of tissues, whereas 50 mg/kg celecoxib caused stomach tissue damage in all mice^27^.

The potential of a highly selective mPGES-1 inhibitor as a substitute of currently used opioids is particularly significant for future postoperative pain management, as opioids are still the mainstay of postoperative analgesia because a truly effective and safe non-opioid analgesic is not available. It should also be significant for future paradigm shift in treatment of other forms of pain.

In general, based on the favorable in vivo data in comparison with opioids, UK4b or another potent and highly selective mPGES-1 inhibitor may serve as a valuable tool (a chemical probe) to further validate mPGES-1 as a promising target for treatment of other forms of pain and other inflammation-related diseases.

## Methods

### Materials and instruments

Compound UK4b was synthesized, purified, and characterized as previously reported^27^. The final sample of the compound had a purity of > 95%. Oxycodone hydrochloride was ordered from Sigma-Aldrich (St. Louis, MO). 1% λ-carrageenan (Sigma-Aldrich, St. Louis, MO) was prepared in saline (0.9% sodium chloride)^47^. Complete Freund’s Adjuvant (CFA) was purchased from Chondrex, Inc. (Woodinville, WA). Male CD-1 mice (28–32 g) and Sprague–Dawley rats (200–275 g) were ordered from Harlan (Envigo, Indianapolis, IN). All the animal experiments were conducted in our animal laboratories within the University of Kentucky’s Division of Laboratory Animal Resources (DLAR) facility (PHS assurance number A3336-01; USDA number 61-R-0002; AAALAC, Intl. Unit # 13). Veterinary care and animal husbandry were provided and supervised by the staff of the DLAR facility. All animals were housed in clean, adequately-sized, plastic cages at 21–22 °C and were allowed ad libitum access to food and water for one week before experiments. They were monitored daily by the study staff and by members of the veterinary staff for general health to detect signs of discomfort due to testing and/or the administration of drugs. All animal experiments were performed in accordance with the Guide for the Care and Use of Laboratory Animals as adopted and promulgated by the National Institutes of Health (NIH), and were in fact also consistent with the ARRIVE (Animal Research: Reporting of In Vivo Experiments) guidelines (https://arriveguidelines.org**)**. The animal procedures used in this study had been approved by the University of Kentucky’s Institutional Animal Care and Use Committee (IACUC)*.*

The Ugo Basile Thermal Plantar Instrument (Stoelting, Wood Dale, IL) was used to measure the paw withdrawal latency (PWL) to a noxious heat source. Paw edema of rats was measured with the Digital Water Plethysmometer (Harvard Apparatus, Cambridge, MA). Mechanical thresholds were measured with von Frey filaments (Health Products for You, Brookfield, CT) using the Up-Down Method. Pharmacokinetics (PK) of UK4b were measured in serum using an Agilent HPLC system 1200 Series G1311A Quaternary Pump, 1100 Series G1329A ALS Autosampler, and 1260 Series G1314B Variable Wavelength Detector. Pharmacodynamic (PD) effects of UK4b on prostaglandin E2 (PGE_2_), prostaglandin I2 (PGI_2_), thromboxane A2 (TXA_2_), prostaglandin F2α (PGF_2α_), and prostaglandin D2 (PGD_2_) were measured using commercially available enzyme-linked immunosorbent assay (ELISA) kits from Cayman Chemical (Ann Arbor, MI): Catalog #514531 for PGE_2_, #512011 for PGD_2_, #515211 for PGF_2α_, #515211 for PGI_2_, and #501020 for TXA_2_ in rat tissue samples; catalog #514531 for PGE_2_ in mouse tissue samples.

### Pre-treatment of carrageenan-induced paw edema and hyperalgesia

Eighty (80) rats were randomly assigned to one of seven treatment groups: untreated, vehicle-carrageenan, UK4b (10 mg/kg, IP)-carrageenan, UK4b (5 mg/kg, IP)-carrageenan, oxycodone (5 mg/kg, IP, -1 h)-carrageenan, oxycodone (5 mg/kg, IP, -4 h)-carrageenan, gabapentin (100 mg/kg, IP, -1 h)-carrageenan, gabapentin (100 mg/kg, IP, -4 h)-carrageenan (*n* = 10 for each group). All animals were acclimated to the Thermal Plantar Instrument for one hour before the initiation of the experiment. One of the two hind paws was randomly selected to serve as the paw being observed for the entire experiment. Prior to administration of any treatment, the baseline (0 h) PWL was assessed by centering the infrared source below the plantar surface of the selected hind paw, starting the source, and then the instrument automatically recorded the latency to paw withdrawal. Two PWL measurements were taken on the same foot at each time point. Baseline hind paw volume was also measured with the Digital Water Plethysmometer at 0 h. At each time point, the hind paw volume was measured twice.

One group of animals (“Untreated”) received no treatment throughout this experiment and PWL was measured at 0, 2, 6, 23, 26, 48, 72, and 96 h to serve as the negative control (no pain). After the baseline assessments of PWL and paw volume, the other 70 rats were randomly administered either vehicle (1 μL/g, IP), UK4b (10 mg/kg, IP), UK4b (5 mg/kg, IP), oxycodone (5 mg/kg, IP), or gabapentin (100 mg/kg, IP). In the UK4b groups and the -4 h oxycodone and gabapentin groups, four hours after treatment, acute paw edema and hyperalgesia were induced by administering an intraplantar injection of 100 μL 1% λ-carrageenan prepared in saline (0.9% sodium chloride) to the previously selected hind paw^47^. In the vehicle and -1 h oxycodone and gabapentin groups, acute paw edema and hyperalgesia are induced similarly, but only one hour after drug treatment, PWL and paw edema were assessed relative to carrageenan injection (2, 6, 23, 48, 72, and 96 h), taking two PWL and two volume measurements on the injected foot at each time point. When PWL and paw edema were not being assessed, animals were kept in their cages with access to food and water ad libitum.

Paw withdrawal latency (PWL in seconds) was graphed relative to time (hours since the carrageenan injection). Paw edema was graphed as the percent increase in paw volume compared to baseline *versus* time relative to the carrageenan injection.

### Post-treatment of carrageenan-induced hyperalgesia

Fifty (50) rats were randomly assigned to one of five treatment groups: carrageenan-vehicle (1 μL/g, IP), carrageenan-UK4b (10 mg/kg, IP), carrageenan-UK4b (5 mg/kg, IP), carrageenan-oxycodone (5 mg/kg, IP), and carrageenan-gabapentin (100 mg/kg, IP) (*n* = 10 for all groups). The PWL of untreated rats from the pre-treatment carrageenan study were used as a negative control in this experiment, as well. Like the pre-treatment experiment, all animals were acclimated to the Thermal Plantar Instrument for one hour before the initiation of the experiment. One hind paw was randomly selected to be the observed paw for the entirety of the experiment. Prior to administration of a treatment, the baseline (-23 h) PWL was assessed as described above. Two PWL measurements were taken on the same foot at each time point.

After the baseline PWL assessment, the 60 rats to be used in this experiment received an intraplantar injection of 100 μL 1% λ-carrageenan prepared in saline (0.9% sodium chloride) to the previously selected hind paw^47^. The animals were returned to their cages with access to food and water ad libitum. Twenty-three hours after administration of carrageenan, PWL was assessed (23 h) to confirm induction of hyperalgesia, and to establish the PWL prior to treatment. The rats were then administered with vehicle (1 μL/g, IP), UK4b (10 mg/kg, IP), UK4b (5 mg/kg, IP), oxycodone (5 mg/kg, IP), or gabapentin (100 mg/kg, IP). PWL was assessed relative to administration of treatment (26, 48, 72, and 96 h), and two PWL measurements were taken at each time point for each rat. PWL (seconds) was graphed relative to time (hours since treatment administered).

### Post-treatment of adjuvant-induced knee joint arthritis

The CFA-induced knee joint arthritis experiment was conducted over 13 days (Days 0–12) and consisted of three periods: the arthritis development period (Days 0–3), the treatment period (Days 3–6), and the follow-up assessment period (Day 7–12). Thirty (30) rats were acclimated to the Thermal Plantar Instrument for one hour on Day 0, and then PWL was assessed as described above. The Day 0 spontaneous pain rating (arthritis score) was also determined, as described previously^48^. In brief, each animal was observed in their home cage and their limb posture was scored on a subjective pain sale, where 0—normal posture; 1—toes are curled; 2—paw is everted; 3—the paw is only partially weight bearing; 4—the paw is non-weight bearing; 5—the rat avoids any contact with the hind paw^48^. After the Day 0 PWL and spontaneous pain scoring was recorded, each rat was briefly anaesthetized with 2% isoflurane inhalation and one knee joint was injected with 100 μL CFA emulsion. Animals were returned to home cages to recover and for the model to develop, where they had ad libitum access to food and water whenever measurements were not being taken.

On Days 1 and 2, spontaneous pain scores were assessed in the morning. After scoring was complete, PWL was assessed. On Day 3, the spontaneous pain score was assessed again in the morning, then the 30 rats were randomly divided into three groups (*n* = 10 for each): vehicle, UK4b (10 mg/kg, IP), and UK4b (5 mg/kg, IP). The treatment was administered, then 2 h after treatment, PWL was assessed. Days 4 through 6 were conducted similarly, with spontaneous pain scoring taking place in the morning prior to treatment, followed by administration of the respective drug, then assessment of PWL two hours after treatment. Finally, on Day 7, the spontaneous pain scores were recorded as normal, followed by assessment of PWL, though no drug was administered on Day 7. On Days 8, 10, and 12 post-CFA injection, the spontaneous pain score was assessed.

The PWL (seconds) was graphed relative to time (days since CFA administration). Spontaneous pain scores were graphed as average score per group *versus* time (days since CFA administration).

### Post-treatment of postoperative pain

Thirty-one (31) mice were randomly assigned to one of four treatment groups: vehicle (5 μL/g, SC) (n = 8), UK4b (20 mg/kg, SC) (n = 7), UK4b (10 mg/kg, SC) (n = 8), and morphine (5 mg/kg, SC) (n = 8). This experiment took place over 14 days. Beginning four days before surgery, the mice underwent acclimation to the Thermal Plantar Instrument for one hour before PWL was assessed. Similarly, four days before surgery, the animals began acclimation for one hour per day to the mechanical sensitization (allodynia) apparatus before assessment. Mechanical sensitization was measured with a series of 8 von Frey fiber filaments (0.008 g (1.65); 0.02 g (2.36); 0.07 g (2.83); 0.16 g (3.22); 0.4 g (3.61); 1.0 g (4.08); 2.0 g (4.31); 6.0 g (4.74); Stoelting, Wood Dale, IL) using a modified up-down method^49^. For the algorithm, each filament is applied to the plantar surface of the hind paw for five times. If the mouse retracts that hind paw at least three times, that is considered a positive response to the filament, and the next applied filament would be weaker than the last. If the animal retracts the hind paw fewer than three times, this is a negative response, and the next stronger filament is applied. Once the first positive response is invoked, four additional fibers are applied in the manner described above. An algorithm is used to then estimate the 50% mechanical sensitization threshold. These acclimation sessions and baseline measurements occurred on Day -4 through Day -1. Surgery was performed under 2% isoflurane anesthesia. A longitudinal incision (5 mm in length) was made on the plantar surface of one hind paw, then a longitudinal incision through the belly of the flexor digitorum brevis muscle was made. The surgery site was then closed with two sutures and the animals were returned to their home cage to recover and for the model to develop, where they had ad libitum access to food and water whenever measurements were not being taken. Two hours after surgery was complete, PWL was assessed, followed by measurement of mechanical sensitization threshold. The animals were then treated once daily with their respective treatment two hours before PWL and mechanical sensitization threshold tests from Days 1 through 7, with the first dose occurring 24 h after surgery was completed. On Day 9, 48 h after the final treatment was administered on Day 7, PWL and mechanical sensitization threshold were assessed to follow-up with the animals after treatment ceased. The PWL (seconds) is graphed relative to time (days since surgery). The PWL (seconds) was graphed relative to time (days since surgery). Mechanical sensitization thresholds were graphed as 50% threshold (grams) *versus* time (days since surgery).

### Pre- and post-treatment of postoperative pain

Twenty-eight (28) mice were randomly assigned to one of four treatment groups: vehicle (5 μL/g, SC) (n = 8), UK4b (20 mg/kg, SC) (n = 7), UK4b (10 mg/kg, SC) (n = 7), and morphine (5 mg/kg, SC) (n = 6). This experiment took place over 14 days. Animals underwent the same acclimation and baseline measurement for PWL and mechanical sensitization threshold as described in the section above on Days -4 to -1. On Day 0, surgery was performed as described above, but each animal received their first treatment two hours prior to surgery (-2 h). Two hours after surgery was complete, PWL was assessed, followed by measurement of mechanical sensitization threshold. Treatment continued once daily from Days 1 through 7. Two hours after drug treatment, PWL and mechanical sensitization threshold are measured. On Day 9, 48 h after the final treatment was administered on Day 7, PWL and mechanical threshold are assessed to follow-up with the animals after treatment ceases. The PWL (seconds) was graphed relative to time (days since surgery). Mechanical sensitization thresholds were graphed as 50% threshold (grams) *versus* time (days since surgery).

### Pharmacokinetic analysis of UK4b in rats

Two doses of UK4b were administered in two groups of rats: UK4b 1 mg/kg (n = 7) or 10 mg/kg (n = 4), IP. Blood samples were collected at various time points after administration. Blood was allowed to clot for 15 min at room temperature before being centrifuged at 10,000 rpm for 15 min. Supernatant serum was removed into a new tube and stored at − 20 °C until analysis by HPLC. After thawing, serum was diluted with 0.3 M HCl and acetonitrile, then centrifuged at 13,100 rpm for 12 min. Supernatant was removed and placed into HPLC vials. Chromatographic analysis was carried out on a Poroshell 120 SB-C18 (120 Å, 2.7 µm, 4.6 × 150 mm) reverse-phase LC column (InfinityLab). The mobile phase used for the isocratic elution consisted of 0.1% formic acid and 70% acetonitrile (v/v) run at a flow rate of 1 mL/min. Absorbance was measured at 350 nm.

### Analysis of the levels of prostanoids in paw tissue of rats

Additional rats (n ≥ 4 per group for each prostanoid) were used to collect necessary paw tissue samples in the model of carrageenan-induced paw edema and hyperalgesia. The collected paw tissue samples were analyzed using the ELISA kits (as noted above) to determine the PGE_2_, PGD_2_, PGF_2α_, PGI_2_, and TXA_2_ levels according to the vendor’s instructions. While each kit requires different steps to purify the sample, the timing of administrating Uk4b and carrageenan, timing of sacrifice, and method of tissue collection remain the same across all five kits. The carrageenan groups receive a subcutaneous (SC) injection of 100 μl 1% λ-carrageenan in saline to the plantar surface of one hind paw at time 0 h (h). In the pre-treatment with UK4b + carrageenan group, rats received an IP injection of UK4b (10 mg/kg) 4 h before carrageenan administration (at − 4 h). In the negative control group, no treatment was administered, but the animals followed the same timing as the other groups. All rats were sacrificed at 26 h, then the skin, muscle, and connective tissue were excised from the plantar and dorsal surface of the paw that received carrageenan.

### Analysis of the PGE_2_ level in paw tissue of mice

Additional mice were used to study the effects of UK4b on PGE_2_ in the mouse model of postoperative pain. The PGE_2_ level in paw tissue was assessed using the ELISA kit (catalog #514,531) according to the vendor’s instruction. For the PGE_2_ assessment, there were four groups of mice: sham (no UK4b or surgery) (n = 8), positive control (vehicle + surgery) (n = 8), surgery + post-treatment with UK4b (n = 6), and pre- and post-treatment with UK4b + surgery (n = 8). The sham group experienced the same set of conditions as the surgery groups, such as the amount of time spent under anesthesia and the surgery site being treated with iodine, except no incision was made to the hind paw. The positive control group received an SC injection of vehicle 2 h before surgery, then once daily on Days 1 and 2 after surgery. The post-treatment surgery group received an SC injection of vehicle 2 h before surgery, then UK4b (20 mg/kg, SC) on Days 1 and 2. The pre- and post-treatment surgery group received UK4b (20 mg/kg, SC) 2 h before surgery and on Days 1 and 2. Animals in all four groups were sacrificed 2 h after the treatment on Day 2. The sutures are removed from the paw, then the entire paw was removed by cutting through the ankle bone. The entire paw was weighed, then flash frozen in liquid nitrogen and stored at − 80 °C before analysis.

### Determination of the intrinsic clearance in liver microsomes and hERG inhibition

The intrinsic clearance of UK4b in liver microsomes (human) was determined by Eurofins Panlabs (St. Charles, MO) through their standard protocol, including measurements at five time-points (0, 15, 30, 45, and 60 min) in order to estimate the intrinsic clearance (CL_int_) and half-life (*t*_1/2_). The inhibitory activity of UK4b at 10 μM against potassium channel hERG was tested by Eurofins Cerep (France) using the standard Cerep protocol.

### Statistical analysis

The comparison in animal behavioral data between different groups of rats was performed by two-way (treatment × time) analysis of variance (ANOVA) with post hoc tests (with repeated measurements over time). Comparisons between groups at individual time points were performed using the student *t*-test. The comparison in prostanoid levels between different groups was performed by one-way (treatment) ANOVA. All statistical analyses were performed using the GraphPad Prism 7.0 software (GraphPad Software, La Jolla, CA). Statistical significance: *p < 0.05; **p < 0.01; ***p < 0.001; ****p < 0.0001.

## Supplementary Information

Supplementary Information.

## Data Availability

The datasets used and/or analyzed during the current study are available from the corresponding author on reasonable request.
