# Supplementary material for: Analgesic effects of a highly selective mPGES-1 inhibitor
Source: Sci Rep. 2023 Feb 27;13:3326. doi: 10.1038/s41598-023-30164-3 (PMC9971260; doi:10.1038/s41598-023-30164-3)
Supplement: Supplementary file 1 — Supplementary Information. [file 41598_2023_30164_MOESM1_ESM.pdf]

Supplementary Information

Analgesic effects of a highly selective mPGES-1 inhibitor

Madeline J. Stewart,[1,2] Lauren M. Weaver,[1,2] Kai Ding,[1] Annet Kyomuhangi,[1,2] Charles D. Loftin,[2] Fang Zheng,[1,2,]* and Chang-Guo Zhan[1,2,]*

[1]Molecular Modeling and Biopharmaceutical Center, College of Pharmacy, University of Kentucky, 789 South Limestone Street, Lexington, KY 40536. [2]Department of Pharmaceutical Sciences, College of Pharmacy, University of Kentucky, 789 South Limestone Street, Lexington, KY 40536

Running Title: Analgesic effects of a selective mPGES-1 inhibitor

Correspondence:
Chang-Guo Zhan, Ph.D.
Director, Molecular Modeling and Biopharmaceutical Center (MMBC)
Director, Chemoinformatics and Drug Design Core of CPRI
University Research Professor
Endowed College of Pharmacy Professor in Pharmaceutical Sciences
Professor, Department of Pharmaceutical Sciences
College of Pharmacy
University of Kentucky
789 South Limestone Street
Lexington, KY 40536
Phone: 859-323-3943
FAX: 859-257-7585
E-mail: zhan@uky.edu

Additional figure for the mouse model of postoperative pain

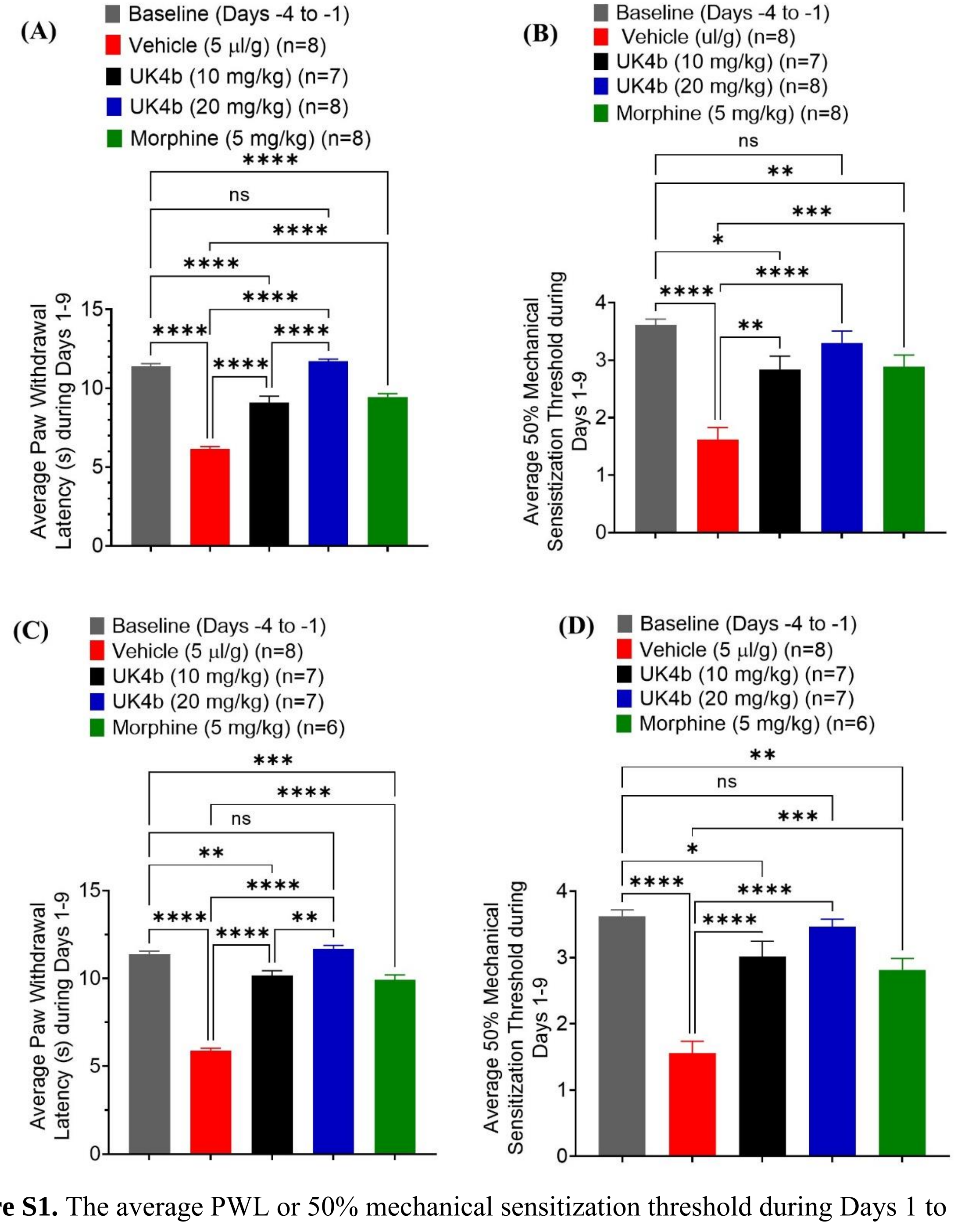

Figure S1. The average PWL or 50% mechanical sensitization threshold during Days 1 to 9 in comparison with the corresponding average baseline during Days -4 to -1 before the surgery in the mouse model of postoperative pain. The averages shown here are calculated by using the time-dependent data shown in **Figure 3** for each group. Statistical significance (one-way ANOVA): * p<0.05; ** p<0.01; *** p<0.001; **** p<0.0001.
